# Potassium depletion induces cellular conversion in the outer medullary collecting duct altering Notch signaling pathway

**DOI:** 10.1038/s41598-020-61882-7

**Published:** 2020-03-31

**Authors:** Anna Iervolino, Federica Prosperi, Luigi R. De La Motte, Federica Petrillo, Manuela Spagnuolo, Mariavittoria D’Acierno, Sabrina Siccardi, Alessandra F. Perna, Birgitte M. Christensen, Sebastian Frische, Giovambattista Capasso, Francesco Trepiccione

**Affiliations:** 1 Biogem S.c.a.r.l., Istituto di Ricerche Genetiche “Gaetano Salvatore”, Ariano Irpino, Italy; 2 Department of Translational Medical Sciences, University of Campania “L. Vanvitelli”, Naples, Italy; 3 Department of Biomedicine, Aarhus University, Aarhus, Denmark

**Keywords:** Nephrons, Nephrons

## Abstract

Potassium depletion affects AQP2 expression and the cellular composition of the kidney collecting duct. This, in turn, contributes to the development of a secondary form of nephrogenic diabetes insipidus and hypokalemic nephropathy. Here we show that after 14 days of potassium depletion, the cellular fraction of A-type intercalated cells increases while the fraction of principal cells decreases along the outer medullary collecting duct in rats. The intercalated cells acquired a novel distribution pattern forming rows of cells attached to each other. These morphological changes occur progressively and reverse after 7 days of recovery on normal rat chow diet. The cellular remodeling mainly occurred in the inner stripe of outer medulla similar to the previously seen effect of lithium on the collecting duct cellular profile. The cellular remodeling is associated with the appearance of cells double labelled with both specific markers of principal and type-A intercalated cells. The appearance of this cell type was associated with the downregulation of the Notch signaling via the Hes1 pathways. These results show that the epithelium of the collecting duct has a high degree of plasticity and that Notch signaling likely plays a key role during hypokalemia.

## Introduction

Potassium depletion (KD) has a major impact on the function of the nephron and the collecting duct. Indeed, in a clinical setting hypokalemia is usually associated with metabolic alkalosis and polyuria^1^. The collecting duct is the main target of KD. Silver *et al*.^2^ reported an upregulation of H^+^-ATPase, H^+^/K^+^-ATPase and AE1 during hypokalaemia. This has been considered the molecular signature of an increased activity of A-type intercalated cells^3^ and to be primarily involved in the pathogenesis of KD related metabolic alkalosis. The origin of KD-related polyuria seems to be multifactorial and involves not only the downregulation of the AQP2 in the principal cells, but also impairing of the medullary interstitial osmolality^4^. These effects on the collecting duct function are similar to the lithium related side effects^5^. These similarities involve also morphological changes in the cellular composition of the collecting duct. Indeed, both lithium administration and KD leads to an increase in the fraction of intercalated cells and a reduction in the fraction of principal cells^6,7^. Recently, by selective ablation of Mib-1, a gene upstream of Notch signaling, Jeong *et al*. were able to reproduce similar changes in the cellular composition of the collecting duct as with lithium administration and KD^8^. These data strongly suggest that the balance of A-type intercalated cells (A-IC) and principal cells (PC) is somehow regulated by Notch signaling.

Since we have previously demonstrated, that the recovery of the lithium-induced changes in the cellular composition of the collecting duct is led by the appearance of a novel cell type that expresses both functional markers of A-IC and PC^6^, here we want to investigate whether similar mechanisms of cellular plasticity could be identified also in association with KD and whether variation in the Notch signaling pathways plays a role in this setting.

Here we showed that the KD-related changes in the cellular composition of the collecting duct are anticipated by the appearance of cells expressing, at the same time, both markers of A-IC and PC, namely the B1 subunit of H^+^-ATPase and AQP2 or AE-1 and AQP4. This phenotype was associated with a dysregulation of the Notch signaling. In conclusion, we show that the KD-induced effect on the cellular morphology of the collecting duct is associated to a demodulation of the Notch signaling pathway.

## Results

### Serum and urinary physiological parameters

**Figure 1:**
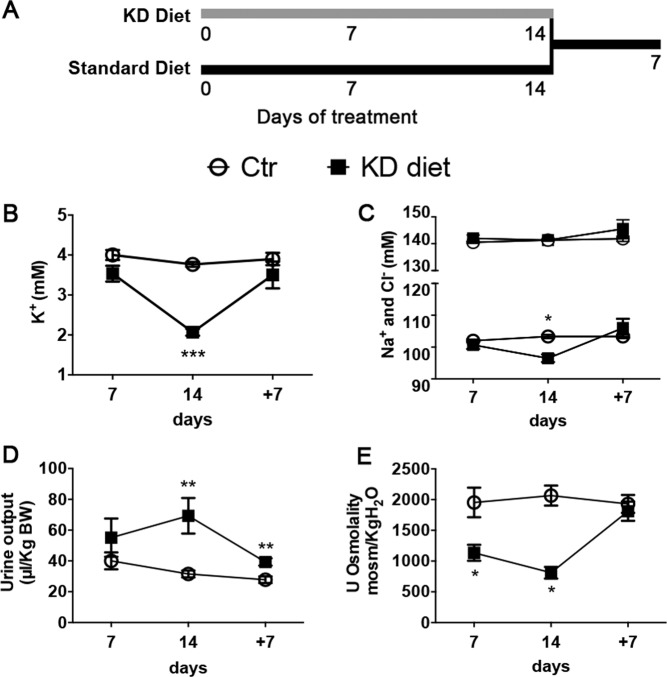
Time course of physiological parameters. Panel A: Male Wistar rats were randomly divided into two groups. The experimental group was fed with 0% K^+^- diet (KD diet, gray line), while the control with the equivalent normal rat chow (Standard diet, black line) for 7 and 14 days (n:6 + 6 per time point). An additional experimental group received normal rat chow after 14 days of KD diet for additional 7 days, while the corresponding controls stayed on control rat chow for a total of 21 days. Panel B: serum K^+^ confirms that KD diet leads to hypokalemia after 14 days. Panel C: serum chloride is significantly reduced, in a setting of normonatremia, after 14 days of KD diet as sign of metabolic alkalosis associated with hypokalemia. Both urine volume (panel D) and urinary osmolality (panel E) are significantly altered by 14 days KD diet. Data are expressed as mean ± se; *p < 0.05, **p < 0.01, ***p < 0.001, two-way ANOVA.

KD diet progressively induces hypokalemia. After 7 days of KD diet, the experimental group presented with a tendency of lower serum K^+^ compared to the control group (3.53 ± 0.19 vs 4 ± 0.12 mM, n:6 + 6, p = 0.07). Hypokalemia developed significantly after 14 days of KD diet and was completely restored after additional 7 days of KD diet withdrawal (recovery phase) (Fig. 1B). The development of hypokalemia was associated with a progressively development of hypochloremia. Indeed, at day 14 of KD diet, in respect to normo-natremia, serum chloride was significantly lower in the experimental group compared to the control (97 ± 1.7 vs 103.8 ± 0.8 mM n:6 + 6 p < 0.05), suggesting that metabolic alkalosis develops together with hypokalemia (Fig. 1C). Hypochloremia was strictly related to hypokalemia, since it resolved after 7 days of recovery together with the restoration of serum potassium level. These electrolytes alterations were in line with a previous published model^6^.

Alteration in potassium homeostasis was paralleled by the development of a progressively severe hypoosmotic polyuria that reverses after 7 days of recovery (Fig. 1D,E) and polydipsia (Table S2B), confirming a direct effect of potassium depletion on renal CD. As previously showed^9^, we confirmed that KD induced a progressive increase in total ammonium excretion (Fig. S1).

### Potassium-induced urinary concentrating defect is associated with an alteration of the cellular composition of the outer medullary CD

**Figure 2:**
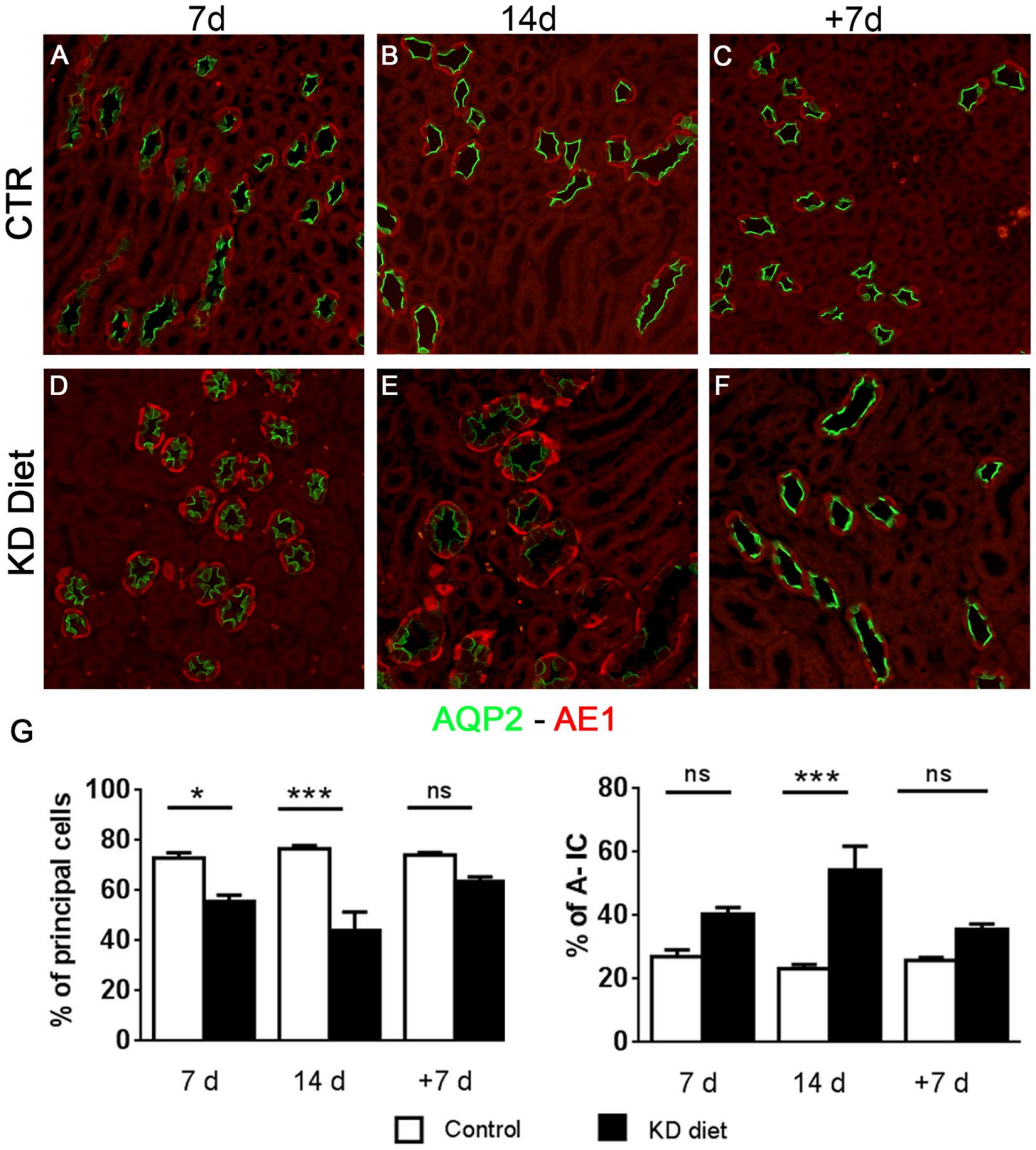
Cellular composition of the CD. Panels A-F show, representative pictures of renal ISOM at different time points of sections double labelled with anti-AQP2 (green, marker of PC) and anti-AE1 antibodies (red, marker of IC). KD diet (**D–F**) induced an increased prevalence of IC compared to controls (**A–C**). In (**G**), the fraction of PC and IC showed that the cellular remodeling of the collecting duct induced by KD started already after 7 days of treatment. Data are expressed as mean ± se; ns = not significant, *p < 0.05, ***p < 0.001, one-way ANOVA.

In order to investigate the cellular composition of the CD after KD diet, we performed a double immunofluorescence labelling for specific markers of PC and A-IC, namely anti-AQP2 and anti-AE-1 antibodies, respectively. In Fig. 2A–F, representative pictures from the inner stripe of outer medulla (ISOM) of rats fed a 7, 14 and 14 + 7R (+7) KD diet showed a progressive increase in the density of A-IC (AE-1 positive cells) with the KD diet. However, 7 days of recovery were sufficient to restore the density of A-IC in the experimental group to similar levels as in the control group. Cellular counting corroborated this observation showing that 14 days of KD diet not only increase the fraction of A-IC, but also reduce the cellular fraction of PCs (Fig. 2G).

**Figure 3:**
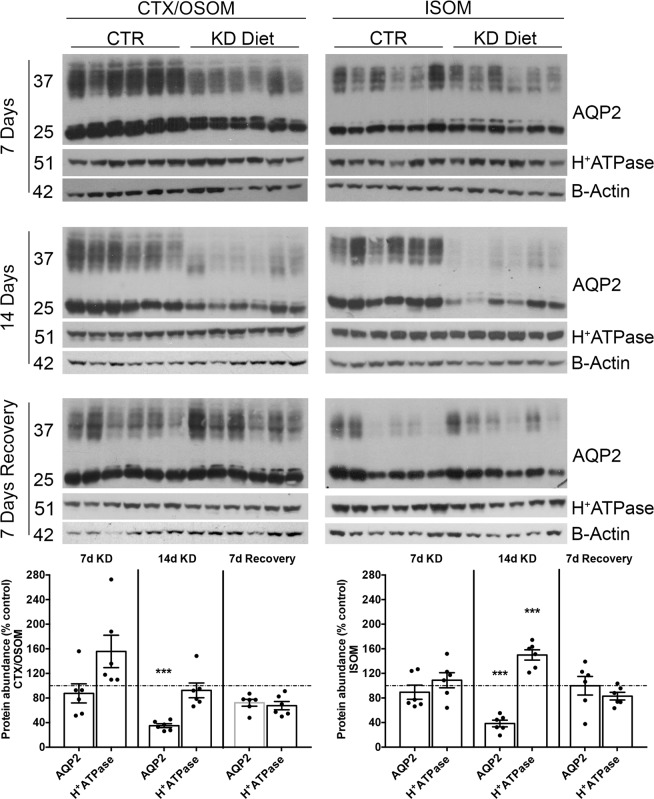
Immunoblotting evaluation of AQP2 and H^+^-ATPase expression. To corroborate the changes in PC and IC expression, immunoblotting from CTX/OSOM and ISOM were carried out at different time points. KD induced a downregulation of AQP2 expression after 14 days of KD and recovered after additional 7 days of normal diet. In the ISOM, the B1 subunit of H^+^-ATPase was upregulated in the experimental group after 14 days of KD diet and remained upregulated after 7 days of recovery. Data are normalized using β-Actin and where expressed as mean ± se of percentage of control, dashed line; n power is 6 vs. 6; ***p < 0.001, unpaired t-test.

To further confirm these findings, we performed immunoblotting of the main specific cellular markers of PC and IC, namely AQP2 and the B1-subunit of the H^+^-ATPase. Quantification of AQP2 expression revealed a statistical significant downregulation in the cortex/OSOM and ISOM both after 14 days of KD diet (Fig. 3). The recovery of AQP2 expression was completed after 7 days of recovery from KD diet. These results confirm previous observations that low potassium induces secondary nephrogenic diabetes insipidus by impairing AQP2 expression^10^. Together with maximal AQP2 protein downregulation after 14 days of KD diet a significant upregulation of the B1-subunit of the H^+^-ATPase was detected in the ISOM (Fig. 3). This is in line with an increased fraction of A-IC in this segment.

### Hypokalemia induces the appearance of cells expressing both IC and PC markers

**Figure 4:**
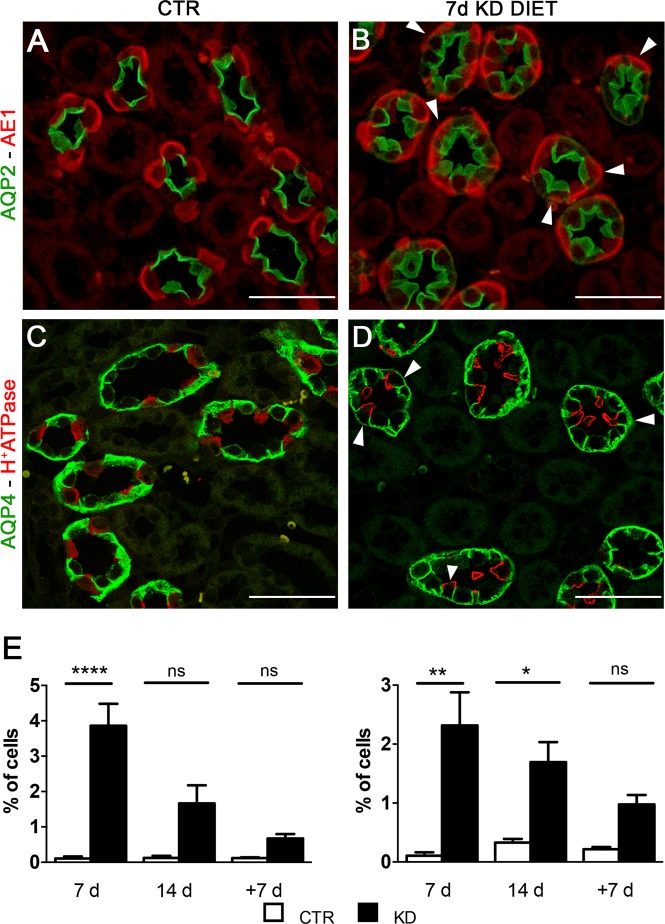
KD diet promotes the expression of AQP2/AE1 and H^+^-ATPase/AQP4 labelled cells. Representative pictures of renal ISOM sections after 7 days of KD labelled with anti-AQP2(green) and anti-AE-1 (red) (**A,B**) or anti-AQP4 (green) and H^+^-ATPase antibodies (red) (**C,D**). White arrows point to double labelled cells in the experimental group (scale bar 20 µm). In (**E**), counting of the double labelled cells after 7 days of KD was performed. Data are expressed as mean ± se; n power is 6 vs. 6; *p < 0.05, **p < 0.01, ***p < 0.001, n.s. not significant; one-way ANOVA.

The effect of hypokalemia on the CD was not limited to the change of the cellular fraction between PC and IC but involved also the distribution pattern of A-IC. Indeed, after 14 days of KD diet A-ICs were organized in doublets and triplets along the CD, losing the typical pattern of interpolation among two principal cells (Fig. 2E). These hypokalemia related morphological changes were similar to the effect of lithium on the CD^6^. In order to investigate whether, the KD effect on the CD was associated with signs of cellular plasticity as seen in the lithium model, we evaluated the presence of cells expressing both specific markers of IC and PC. By performing a double labelling with anti-AQP2 and anti-AE1 antibodies on 2 µm thick sections, we identified some cells unusually expressing both markers (Figs. 2 and 4A,B). The presence of this novel cell type was also identified, when the sections were probed with anti- B1-subunit H^+^-ATPase/anti-AQP4 antibodies (Fig. 4C,D). Similar to the lithium model, this novel cell type was mainly identified in the ISOM. Cell counting of this cell type revealed a higher percentage after 7 days compared to 14 days of KD diet suggesting that the double labelled cells could represent an intermediate state of the PC trans-differentiation into IC. After 7 days of recovery from KD diet, only few double labelled cells could be still identified in the experimental group (Fig. 4E).

### Hypokalemia induces dysregulation of Notch signalling pathway

**Figure 5:**
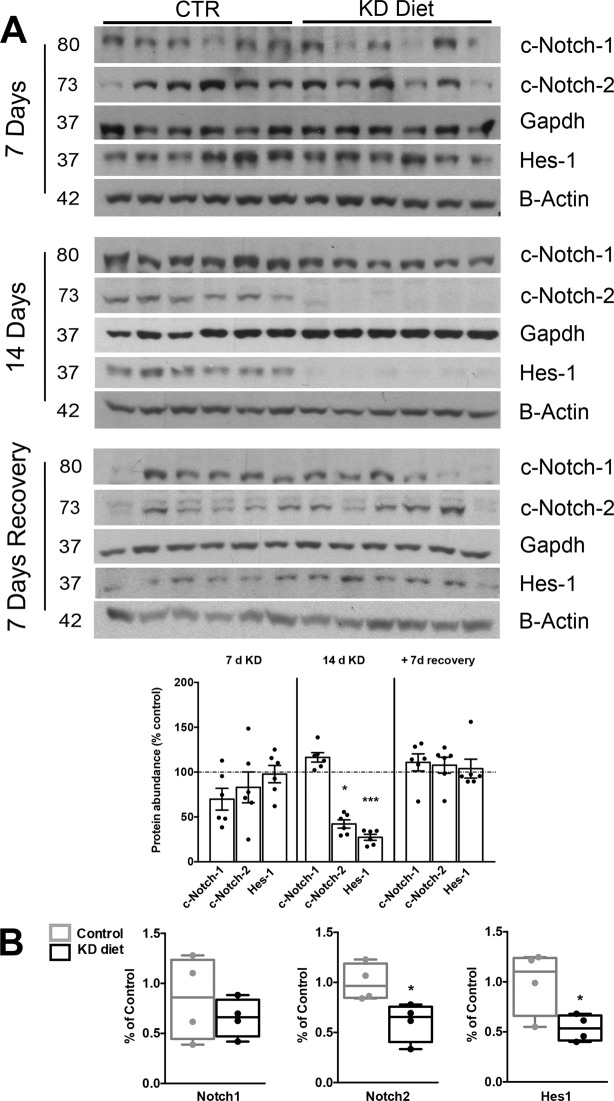
KD diet suppress Notch signaling via Hes1 pathway. Western blot analysis of the cleaved Notch1 (c-Notch1), cleaved Notch2 (c-Notch2) and its effector molecule Hes1 after 7 and 14 days of KD and after additional 7 days of recovery from ISOM samples (**A**). KD diet (14 days) is associated with a downregulation of protein abundance of c-Notch2 and Hes-1. Data are expressed as mean ± se; n: 6 vs. 6, *p < 0.05 (unpaired t-test). RT-qPCR from ISOM (**B**) for Notch1, Notch2 and Hes1. After 10 days, KD induces a downregulation of Notch2 and Hes1 in the ISOM. Data are expressed as mean ± se; n: 5 vs. 5, *p < 0.05, unpaired t-test., *** p < 0.001.

Notch signaling pathway has been proven to be crucial for the proper cellular composition of the CD and in particular for the PC differentiation^8^. In order to investigate whether the dysregulation of the Notch signaling pathways is involved in KD - induced cellular changes of the CD, we tested the expression levels of key elements of this pathway. In Fig. 5A, we showed the protein abundance of the cleaved Notch1 and Notch2 form. These activated forms are nuclear trigger of the downstream gene Hes-1^11^. In the ISOM, after 14 days of KD, Notch2 expression was significantly downregulated and so for its downstream effector Hes-1. Hes-1 plays a key role in the development of PCs along the collecting duct^12^. After additional 7 days of recovery, in which the rats were maintained at control diet, no variation of Notch-signaling elements were observed among the groups. The dysregulation of Notch signaling pathway was anticipated by the downregulation of mRNA level of Notch2 and Hes-1 after 10 days of KD (Fig. 5B). At this time point, no variation at protein level was detected in Notch2 and Hes-1 (Fig. S2). Parallel evaluation of FOXI-1, a transcription factor associated to intercalated cells differentiation, reveals an upregulation of FOXI-1 mRNA level in rats fed with KD for 14 days. FOXI-1 level was still significant upregulated after 7 days of recovery (Fig. S3). All together, these data prove that the KD-induced cellular changes of the medullary CD are associated to a downregulation of the Notch signaling via Hes1.

## Discussion

Hypokalemia severely targets the renal function and morphology^1^. Chronic hypokalemia leads to renal hypertrophy and to the so called hypokalemic nephropathy, a type of interstitial nephritis affecting mainly the medullary segments of the kidneys^13,14^. Tubular remodeling already starts after 3 days of KD diet and involves mainly the proximal and distal convoluted tubule, and the CD as demonstrated by optical clearing techniques^15^.

The distal nephron and collecting duct include several cell types and they are highly heterogenic in the function and morphology^16^. However, we and other have recently demonstrated by a fate mapping approach that these cells type share a common precursor and so, at least partly, share the same lineage differentiation path^17,18^. Recently additional evidences in this sense were produced by the analysis of the transcriptome of the single cells of the CD^19^. These evidences raise the possibility that fully differentiated cells of adult CD could be reprogrammed and change their phenotype either to be resistant to an applied toxic agent or to accomplish specific new functions. We have provided evidence of cellular plasticity for the morphological changes underlying the effect of lithium in the CD^6^. Indeed, in this case, we were able to show that cells, acquiring a double phenotype of both PC and A-IC, lead the recovery from the toxic effect of lithium in the CD^6^. Since the KD diet induces similar morphological changes along the CD as in the lithium model, in particular an increase in the fraction of A-IC and decrease in the fraction of PC as already showed^2,10^, we tested here whether double labelled cells could drive this phenotype. Here we confirmed that KD diet, as in the lithium model, induced a downregulation of the fraction of PC as proven both by cell counting in parallel with a decrease in AQP2 expression as well as an increased fraction of A-IC, together with upregulation of the B1 subunit of the H^+^-ATPase. As in the lithium study, we identified here some cells expressing both markers of A-IC and PC in the combination of AE1/AQP2 and B1 subunit of H^+^-ATPase/AQP4, respectively. This cell type was particularly abundant at 7 days of KD diet, which was the earliest examined time point. Their location, as showed previously in the lithium model, was mainly localized in the ISOM suggesting they could potentially be an element of transition between PC to A-IC. The phenotype of this cell type is very similar to the ones showed by ParK EY *et al*. in the recovery phase of hypokalemia induced-cellular remodeling of CD^20^. These double labelled cells are very rare in the control state, but they can expand during state of disease like lithium or KD diet and are considered a transition phase between PC and IC^19^.

How hypokalemia and lithium treatment can lead to similar alteration along the CD remains still unsolved. However, beside significant differences in the overall acid-base homeostasis (hypokalemia is related to metabolic alkalosis and lithium to metabolic acidosis), both KD and lithium enriched-diet are able to strongly stimulate ammonia-genesis^5,21^. Since the CD in the ISOM is the major site for ammonium secretion also during hypokalemia^22^ we hypothesize here a potential direct role of ammonium excretion in CD remodeling. Recently, alfa ketoglutarate, a metabolite of ammoniagenesis, has been identified as crucial paracrine regulator of tubular remodeling. In SPAK KO mice, indeed, together with a hypoplasia of the DCT, an increase urinary ammonium excretion associated with tubular cells remodeling is reported. However, in SPAK KO mice, there is an increased fraction of PC and a decreased number of A-IC sustained by the Notch signaling pathway activation^23^, exactly the opposite in respect to the cellular changes secondary to hypokalemia and lithium. In this way the association between increased urinary ammonia and cellular remodeling seems to be “thigh”, but how this could affect the regulation of Notch signaling deserve further investigation. Here, we observed a progressive dysregulation of the Notch signal from day 7 of KD (no alteration) to day 10 of KD, mRNA level altered, and finally day 14 of KD when alteration at protein level is detectable. This is the time frame in which the large number of double labelled cells is identifiable, suggesting that dysregulation of Notch signal facilitates the appearance of the converting cells as in a recent study elegantly demonstrated^12^. Indeed, inducible suppression Notch signaling via Hes1 led to conversion of PC to A-IC^12^. In conclusion, we showed here that KD diet affects the cellular composition of the CD promoting the appearance of cells expressing both markers of A-IC and PC. These morphological alterations of the composition of the collecting duct are associated to a dysregulation of the Notch signalling pathway via Hes1.

## Materials and methods Animal study

Male Munich-Wistar rats were stabled at 23 °C and maintained at 12-h day-light cycle on a corn cob bedding. Rats were treated according to the following protocols: (1) 0% potassium or control diet for 7 days (n:6 + 6), (2) 0% potassium or control diet for 14 days (n:6 + 6), (3) 0% potassium diet for 14 days followed by 7 days of normal diet while control rats normal diet for 21 days (n:6 + 6) (Fig. 1A). Group allocation was randomly assigned based on BW at baseline (Table S1A). At the end of each protocol the rats were housed individually in metabolic cages for the last 3 days and physiological parameters were measured the last 24 h. All the procedures involving animals were performed according to the Italian Ministry of Health decree nr 100/2006 and later decree 26/2014. *In vivo* experiments were also approved by the local Animal Ethics Committee (CESA) of Biogem (Ariano Irpino, Italy) with ID 4613 and 7917.

### Urine and Blood analysis

Blood was collected through the aorta before perfusion. Serum concentrations of sodium, potassium, and chloride were determined by ISE 8000 electrolyte analyser. Urine collection was collected under paraffin oil. Urine osmolality was measured by freezing point depression Osmometer 3320 (Advanced Instrument, Inc). Urinary ammonium was evaluated by acid-base titration (785 DMP Titrino, Metrohm).

### Immunohistochemistry

Rats were anesthetized with isoflurane and the left kidney was collected for protein and mRNA evaluation. Then, 4% paraformaldehyde was perfused anterograde through the abdominal aorta as previously described^24^.

Immunofluorescence was performed as previously described^6^. Briefly, 2 µm thick paraffin- embedded sections were cut at a microtome (Microm HM335E Bio Optica, Milan, Italy). After target retrieval with TEG buffer pH 9.2, primary antibodies were incubated overnight at 4 °C. Secondary antibodies were incubated for 1 h at room temperature. Stained sections were mounted with fluorescent mounting medium (Dako, CA, United States). For double labelling immunofluorescence, the following primary antibodies were used: goat anti-AQP2 (C-17; Santa Cruz, Dallas, TX, USA) (dilution 1:1000), rabbit polyclonal anti-AQP4 (dilution 1:2000; AQP-004 Alomone Laboratories), anti-mouse H^+^-ATPase (dilution 1:100; sc-55544, Santa Cruz, Dallas, TX, United States) and rabbit polyclonal anti-AE-1 antibody (dilution 1:25; 758AP)^25^. The following secondary antibodies were used: anti-goat Alexa Fluor 488 (dilution 1:800; Invitrogen, CA, United States), anti-rabbit CY3 (dilution 1:500; A10520, Invitrogen, CA, United States) and goat anti- mouse Alexa Fluor 488 (1:800; Invitrogen, CA, United States)^24^ were used. Zeiss spinning disk Axio Observer Z1 confocal microscope (Zeiss, Oberkochen, Germany) was use for the acquisition of images.

### Cell counting and identification of double labelled cells

Cell counting of the CD was performed as described before^26^. Briefly, PCs were identified as AQP2+ AE1−; the A-IC as B1 subunit of H^+^-ATPase + AQP2−. The counting was performed in the inner stripe of outer medulla (ISOM), since the largest morphological effect of KD diet was seen in this renal zone. Images were acquired at 20X objective with a Zeiss spinning disk Axio Observer Z1 confocal microscope (Zeiss, Oberkochen, Germany). Ten random pictures per rat were acquired for counting. The average total number of counted cells per rat was as following: at 7 days of treatment 370 ± 8 vs 436 ± 10, control vs KD, respectively; at 14 days of treatment 384 ± 5 vs 466 ± 27, control vs KD, respectively; at 7 days of recovery 365 ± 7 vs 394 ± 9 control vs KD, respectively. Image-j software was used for cells quantification. Furthermore, double-labeled cells were identified, as previously described^5^, by expressing apical AQP2+ and basolateral AE1+ or apical B1 subunit of H^+^-ATPase+ and basolateral AQP4+ staining. Cells located at the tubule edges and/or labelled with a dyscontinuous fluorescent signal were not counted. Counting of double-positive cells was performed on sections labeled for either AQP2/AE1 and the percentage of double labelled cells was calculated by the total counted nucleated cells of the CD. The nucleus was morphologically identified, in case of uncertainty picture at Differential Interference Contrast were acquired to a better definition of nuclei and cell membrane (Fig. S4).

### Immunoblotting

Immunoblotting performed as previously described^27^. Briefly, left kidney was divided in cortex/OSOM, ISOM, and IM. Here, we used only samples from CTX/OSOM and ISOM as indicated in the figure legend. Purity of the ISOM dissection was tested by evaluating NCC mRNA abundance as showed in Fig. S5. Tissues were homogenized with a Tissue Lyser (Qiagen) in Lysis buffer (Sucrose 0,3 M, Imidazole 25 mM, EDTA 1 mM, PMSF 1 mM) with protease and phosphatase inhibitor cocktails (Complete Protease Inhibitor Cocktail, (sc-29130, Santa Cruz; PhosSTOP, Roche)). Total protein concentration was measured by BCA kit (Biorad Protein Assay). SDS-PAGE was performed on 12.5% gel and proteins were then transferred to PVDF membranes (Invitrolon PVDF, Invitrogen). The membranes were probed with rabbit anti-AQP2 (dilution 1:1000; 7661AP)^28^, mouse anti-B1/B2 subunits of H^+^-ATPase (dilution 1:1000; sc-55544, Santa Cruz), rabbit anti-Notch1 (cleaved N-terminal) (dilution 1:500; 100-401-407, Rockland), Rabbit anti-Nocht2 (cleaved N- terminal) (dilution 1:400; 100-401-408, Rockland) was validate against spontaneously negative liver tissue and liver cell line as showed in Fig. S6, mouse anti-Hes1 (1:300; sc-166410 (E-5), Santa Cruz) and GAPDH (dilution 1:20000; GeneTex 100118), Blots were incubated with HRP conjugated secondary antibodies, according to the species of the primary antibodies (Amersham) and then developed using ECL substrate (Pierce). Image-j was used to quantify single band intensity. For AQP2, the glycosylated and non-glycosylated bands were quantified together.

### RNA Extraction and q-PCR

Total RNA was isolated from the kidney tissues using TRIsure reagent (BIOLINE, A Meridian life Science® Company, WilfongRdMemphis, TN, United States) and 1 μg of RNA was reverse- transcribed by Quantitec reverse transcription kit (Qiagen, Milan, Italy) according to the manufacture’s instruction. The q-PCR was performed with Power PCR Master Mix 16 (Applied Biosystems, Waltham, MA, United States) according to the manufacturer’s instruction and the primers listed in Table 1 were used. PCR setting was: denaturation step at 95 °C for 10 minutes, 40 cycles of denaturing at 95 °C for 10 s, annealing and extension at 60 °C for 1 minute then 95 °C for 15 s, 60 °C for 15 s and 95 °C for 15 s was performed on a 7900HT system (Applied Biosystems, Waltham, MA, United States). Data were analyzed using the ΔΔCt method, normalizing on GAPDH. Dissociation curves are showed in Supplementary Fig. S7.

**Table 1:** List of primers used to perform q-PCR Table 1.

Gene	Sequence	Efficiency
Notch1 Forward	TCCCCTACAAGATCGAAGCC	0.84
Notch1 Reverse	CCCACAAAGAACAGGAGCAC	
Notch2 Forward	TGTGGTCACTGATCCTTCCC	0.82
Notch2 Reverse	TCACATCCCTCCTCCCAAAC	
Hes1 Forward	GTGGGTCCTAACGCAGTGTC	0.98
Hes1 Reverse	GTCAGAAGAGAGAGGTGGGCTA	
Foxi1 Forward	CGTCTCACACTGAGCCAGAT	1.15
Foxi1 Reverse	TCACAGTTGGGGTCCAGAGT	
NCC Forward	ATGTGGTACCCGCCTACGAA	1.11
NCC Reverse	GTGGCTACCTTCCTGCTTGA	
GAPDH forward	CATGGCCTTCCGTGTTCCTA	0.94
GAPDH Reverse	CCTGCTTCACCACCTTCTTGA	

### Statistics

Values are represented as mean ± standard errors. Data were analyzed by Student’s t-test or one- or two-way ANOVA as indicated in the figure legend. P-value < 0.05 was considered significant.

## Supplementary information

Supplementary Data.

## Data Availability

All data generated or analysed during this study are included in this published article and its Supplementary Information files.
