# Supplementary material for: Potassium depletion induces cellular conversion in the outer medullary collecting duct altering Notch signaling pathway
Source: Sci Rep. 2020 Mar 31;10:5708. doi: 10.1038/s41598-020-61882-7 (PMC7109050; doi:10.1038/s41598-020-61882-7)
Supplement: Supplementary file 1 — Supplementary Data. [file 41598_2020_61882_MOESM1_ESM.pdf]

Potassium depletion induces cellular conversion in the outer medullary collecting duct altering Notch signaling pathway

Anna Iervolino[1*], Federica Prosperi[1*], Luigi R. De La Motte[1], Federica Petrillo[1], Manuela Spagnuolo[2], Mariavittoria D'acierno[1,2], Sabrina Siccardi[1,2], Alessandra F. Perna[2], Birgitte M. Christensen[3], Sebastian Frische[3], Giovambattista Capasso[1,2], Francesco Trepiccione[1,2].

[1] Biogem S.c.a.r.l., Istituto di Ricerche Genetiche "Gaetano Salvatore", Ariano Irpino, Italy
[2] Department of Translational Medical Sciences, University of Campania "L. Vanvitelli", Naples, Italy
[3] Department of Biomedicine, Aarhus University, Aarhus, Denmark

* These authors equally contributed

MS is currently at Curie Institute, PSL Research University, Sorbonne University, CNRS UMR 3244, F-75005 Paris, France

Potassium depletion induces cellular conversion in the outer medullary collecting duct altering Notch signaling pathway

Anna Iervolino, Federica Prosperi, Luigi R. De La Motte, Federica Petrillo, Manuela Spagnuolo, Mariavittoria D'acierno, Sabrina Siccardi, Alessandra F. Perna, Birgitte M. Christensen, Sebastian Frische, Giovambattista Capasso, Francesco Trepiccione.

4A 7d

CTX/OSOM

KD Diet | CTR

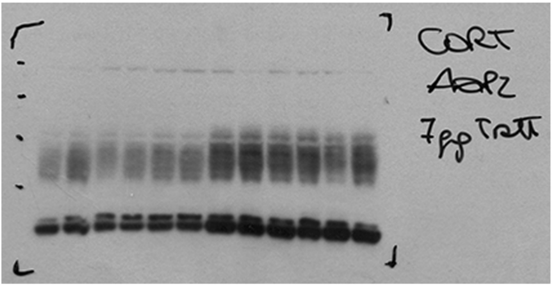

KD Diet | CTR

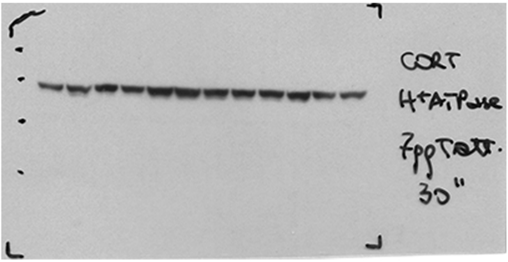

KD Diet | CTR

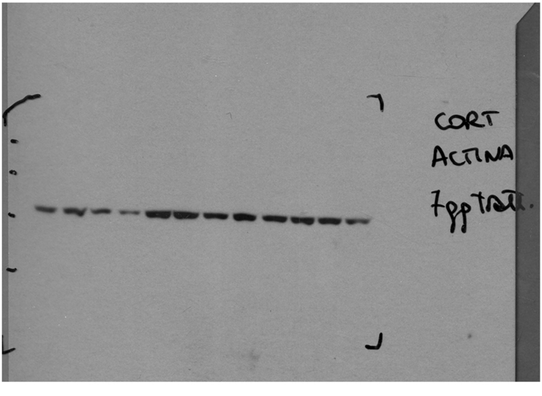

4A 7d

ISOM

KD Diet CTR

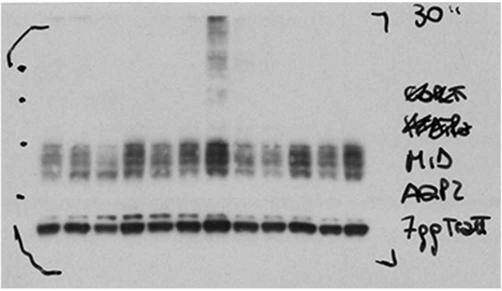

KD Diet CTR

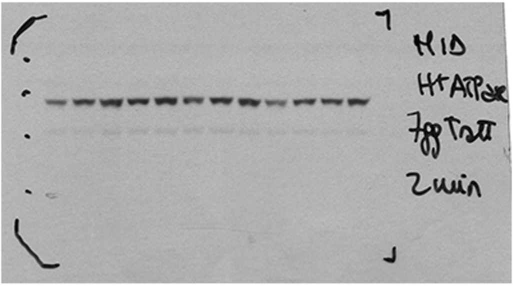

KD Diet CTR

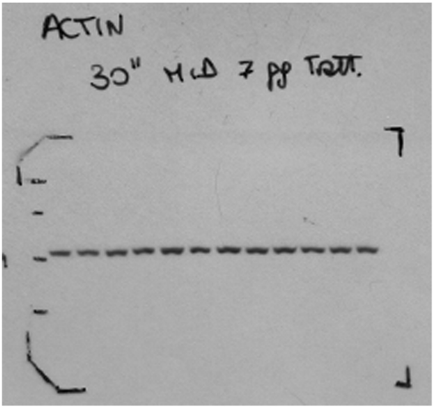

4A 14d

CTX/OSOM

KD Diet | CTR

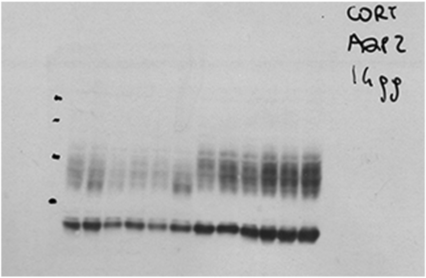

KD Diet | CTR

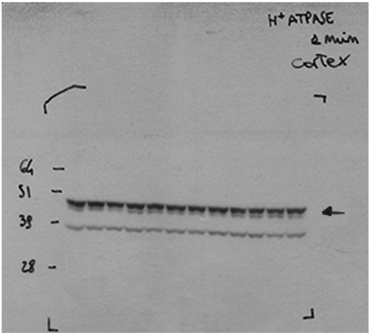

KD Diet | CTR

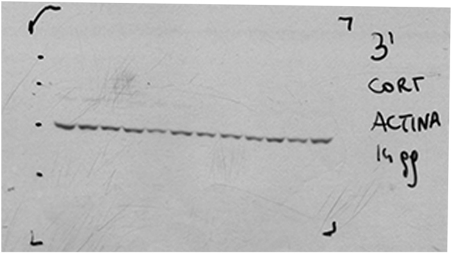

4A 14d

ISOM

KD Diet CTR

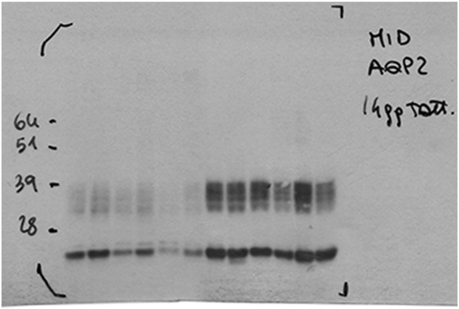

KD Diet CTR

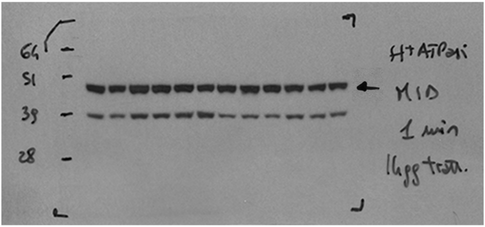

KD Diet CTR

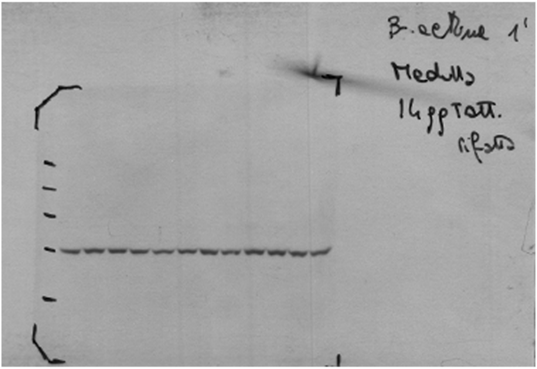

4A +7r

CTX/OSOM

CTR KD Diet

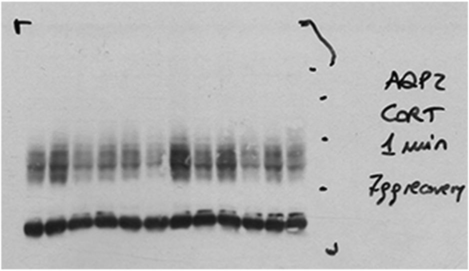

CTR KD Diet

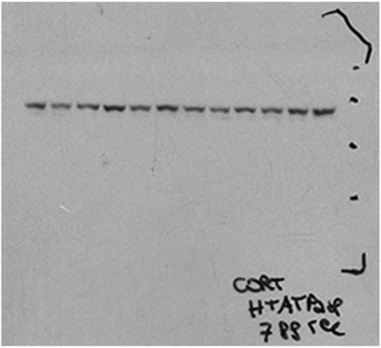

CTR KD Diet

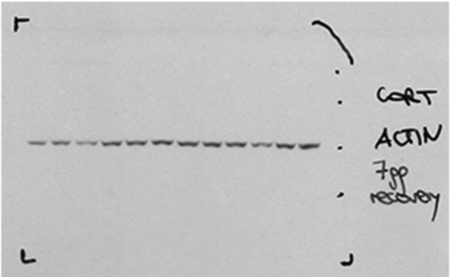

4A +7r

ISOM

CTR KD Diet

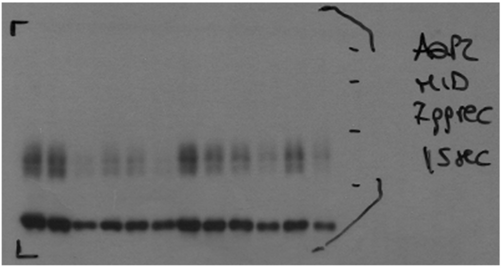

CTR KD Diet

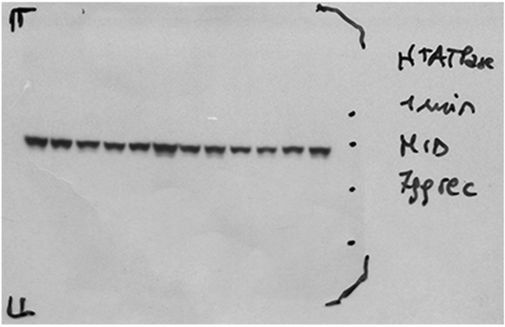

CTR KD Diet

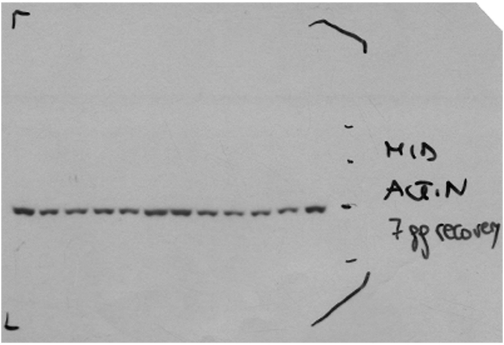

6A 7d

ISOM

KD Diet CTR

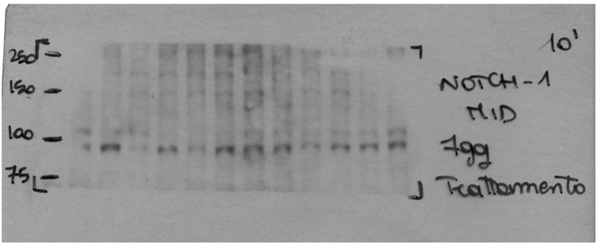

KD Diet CTR

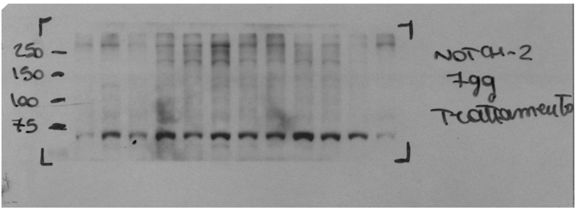

KD Diet CTR

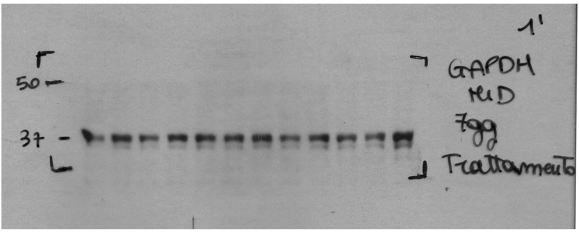

KD Diet CTR

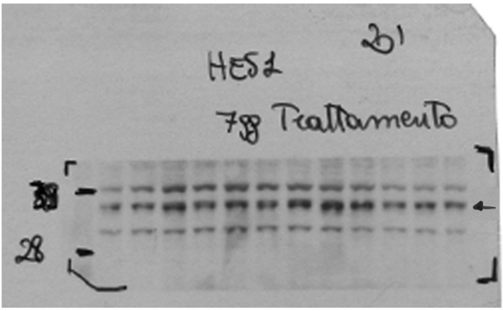

KD Diet CTR

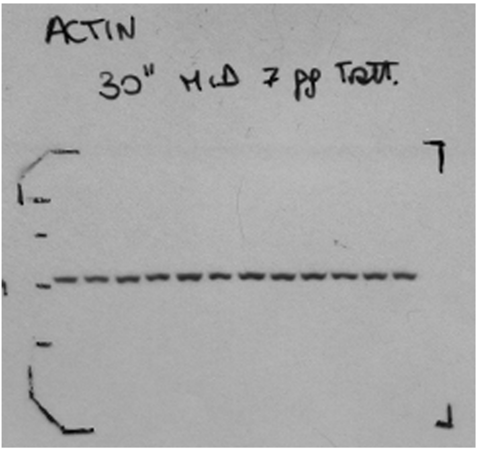

6A 14d

ISOM

KD Diet CTR

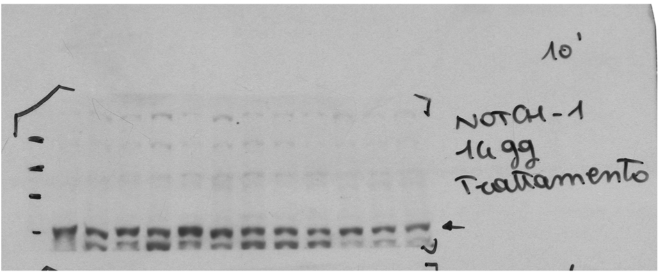

KD Diet CTR

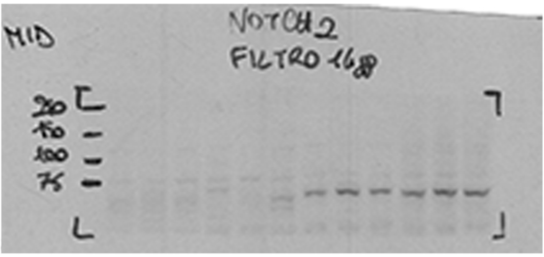

KD Diet CTR

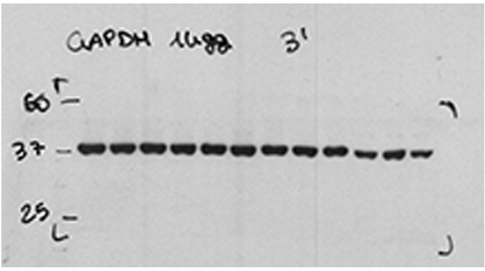

KD Diet CTR

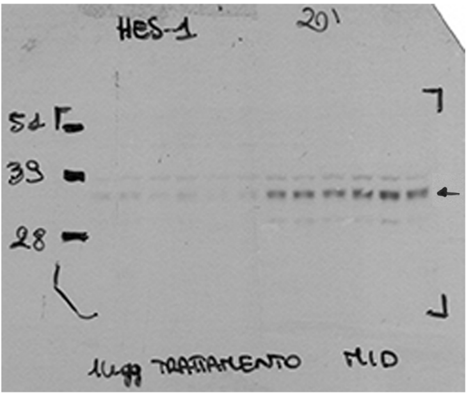

KD Diet CTR

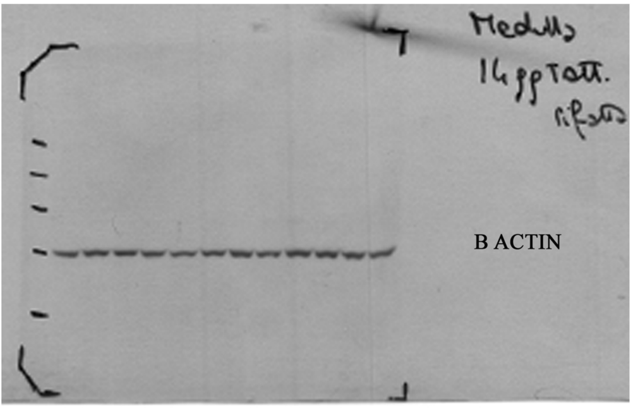

6A +7r

ISOM

KD Diet CTR

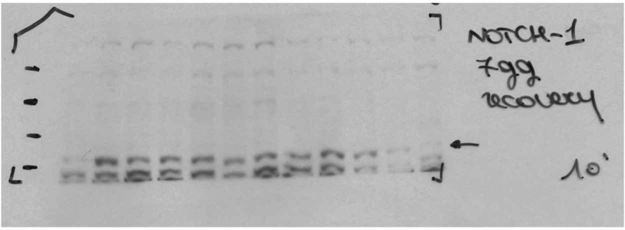

KD Diet CTR

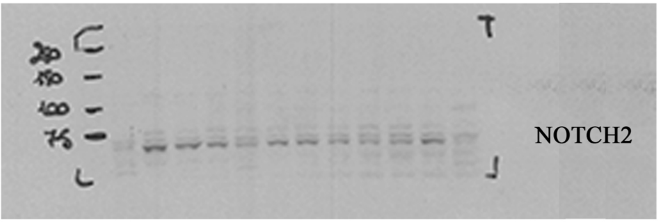

KD Diet CTR

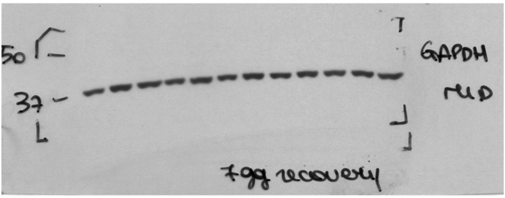

KD Diet CTR

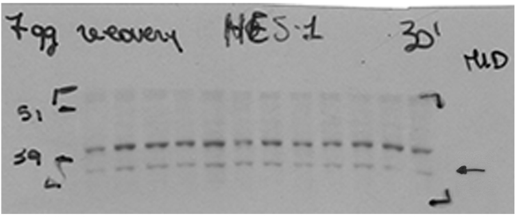

KD Diet CTR

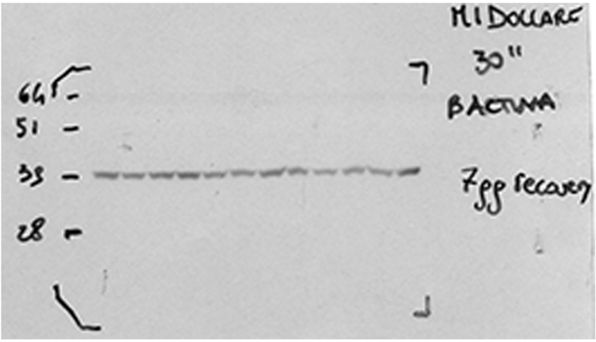

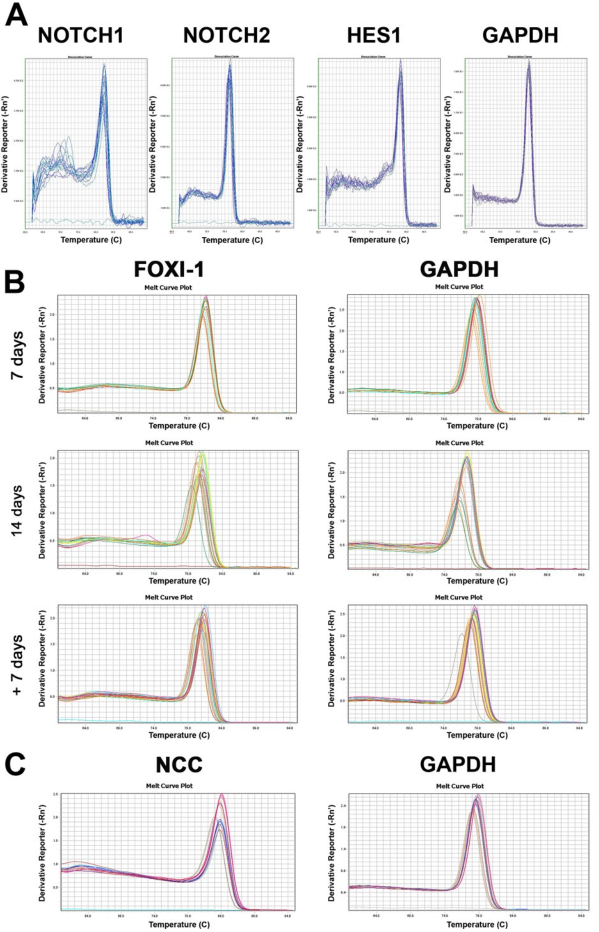

Fig.S7: Melt Curve plot

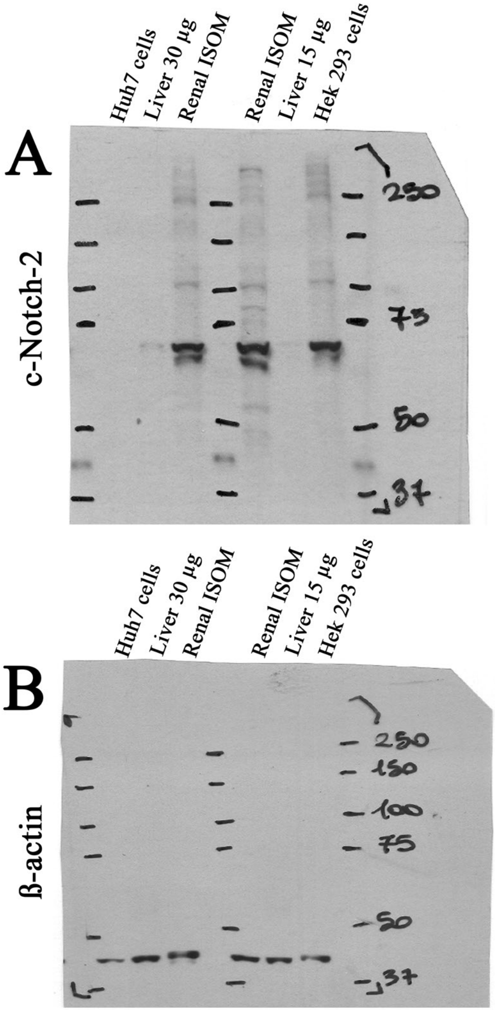

Fig.S6 Validation of anti-c-Notch2 antibody
In order to validate the selectively of anti-Notch2 antibody, tissues from renal ISOM, liver (naturally negative tissue), Hek 293 cells (Human Embryo Kidney cells) and Huh7 cells (hepatocyte derived cellular carcinoma cell line) was used. Anti-c-Notch2 antibody detects a band at the expected size (70 KD) in ISOM and Hek293 cells. As expected in liver no bands were detected by the antibody when 15 µg of tissue were used. A fainth band was detected when 30 µg of liver tissue were loaded, likely due to non-hepatocyte component of the tissue, since in Huh7 cells (an hepatocyte enriched cell line) no band was detected. In panel B, the same membrane was probed with an anti-ß-actin antibody, showing protein loading also in c-Notch-2 negative bands.

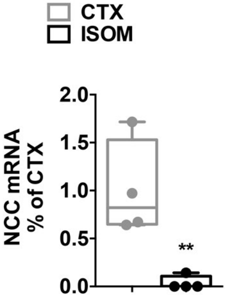

Fig.S5 qPCR evaluation of NCC mRNA in ISOM
In order to assess the purity of the ISOM tissue isolation, mRNA expression of NCC (NaCl co-trasporter, a protein selectively expressed in the CTX) was evaluated in the cortex (CTX, grey dot) and inner stripe of outer medulla (ISOM, black dot).

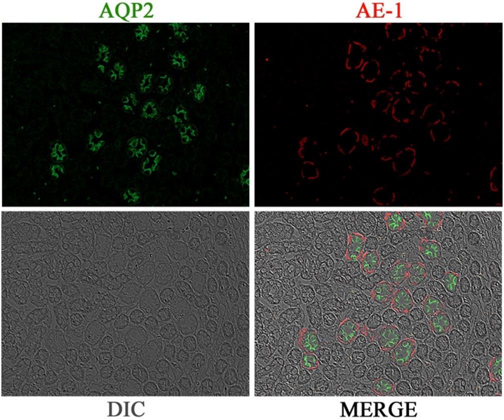

Fig.S4 Evaluation of double labelled cells
Representative pictures from ISOM of a 7d KD-treated rat double labelled with an anti-AQP2 (green) and anti-AE1 (red) antibodies. Differential Interference Contrast (DIC) was used to establish the cell borders and the nucleus in case of uncertainty. All three channels together are represented in the MERGE pannel.

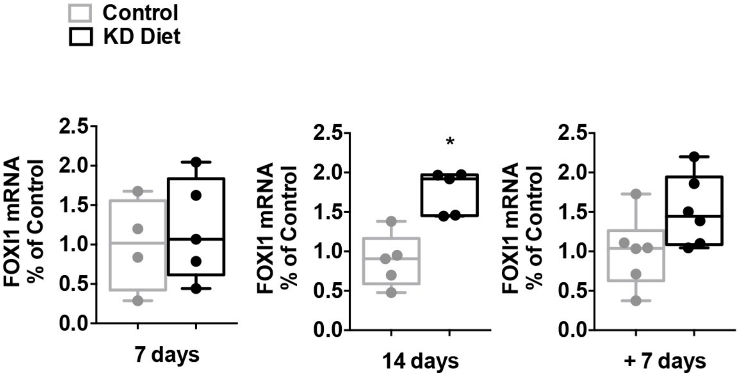

Fig.S3 qPCR evaluation of Foxi-1 mRNA.
qPCR evaluation of Foxi-1 mRNA of ISOM from rats fed with KD diet (black dots) and control diet (grey dots) after 7, 14 days of treatment and additional 7 days of recovery. A significant increase in Foxi-1 level was detected after 14 days of KD-treatment. Data are expressed as mean ± se; n: 4 vs. 4 at 7d, 5 vs 5 at 14d; 6 vs 6 at +7d of recovery; * is for $p < 0.05$, unpaired t-test.

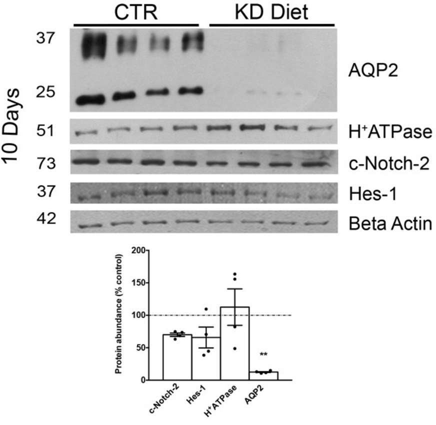

Fig.S2 Notch2, Hes-1, AQP2 and H+ATPase expression after 10 days of KD
Immunoblotting of ISOM from Control and 10 days KD-treated rats were carried out to assess the abundance of c-Notch2/Hes-1 and markers of principal (AQP2) and Intercalated cells (H+ATPase). Although AQP2 was severly downregulated, no significant chages were detected in Notch2/Hes-1. Data are normalized using β-Actin and where expressed as mean ± se of percentage of control (dashed line); n power is 4 vs. 4; * $p < 0.05$, unpaired t-test was used.

Supplementary Tab-1A

BSL	KD				Control					
**BW (gr)**	258	±	7	(18)	264	±	6	(18)	p-value	n.s.

BW evaluation and random group allocation before starting KD-diet

Supplementary Tab-1B

Physiological parameters of rats at each studied time point.

**Day 7**	**KD**				**Control**					
BW (gr)	290	±	10,77	(6)	278	±	10	(6)	p-value	n.s.
WI µl/min/kg	85	±	11	(6)	69	±	6	(6)	p-value	n.s.
**Day 14**	**KD**				**Control**					
BW (gr)	273	±	15	(6)	321	±	15	(6)	p-value	0.04
WI µl/min/kg	103	±	14	(6)	59	±	3	(6)	p-value	0.01
**Day +7**	**KD**				**Control**					
BW (gr)	298	±	14	(6)	335	±	6	(6)	p-value	0.03
WI µl/min/kg	62	±	2	(6)	50	±	2	(6)	p-value	0.004

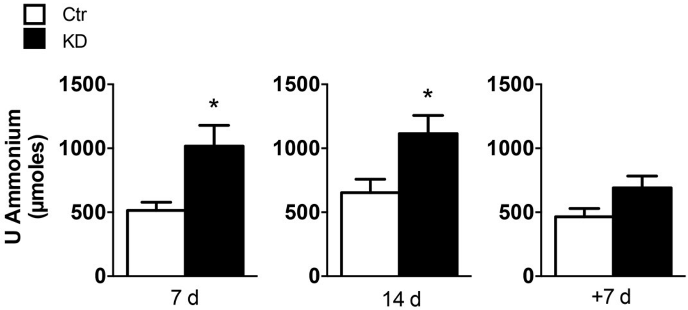

Fig.S1: Urinary ammonium excretion
24h urinary ammonium excretion in rats fed with a K^+ depleted diet (KD, black bar) and in controls fed with normal diet (Ctr, white bar). KD increases urinary ammonium excretion after 7 and 14 days of treatment. Data are expressed as mean ± se; * $p < 0.05$, unpaired t-test.
